# Glasgow Coma Scale (GCS) Score and Full Outline of UnResponsiveness (FOUR) Score in Predicting Outcomes in Patients Admitted to the Medical Intensive Care Unit of Patan Hospital

**DOI:** 10.7759/cureus.108684

**Published:** 2026-05-11

**Authors:** Rupesh Karna

**Affiliations:** 1 Internal Medicine, Koshi Hospital, Biratnagar, NPL

**Keywords:** auroc, four score, glasgow coma scale, in-hospital mortality, survival

## Abstract

Background

Accurate assessment of consciousness is essential for prognostication in critically ill patients. The Glasgow Coma Scale (GCS) is widely used but has limitations in intubated patients and does not assess brainstem reflexes or respiratory pattern. The Full Outline of UnResponsiveness (FOUR) score addresses these limitations. This study compared the predictive performance of GCS and FOUR scores for in-hospital mortality among non-traumatic medical intensive care unit (MICU) patients at Patan Hospital.

Methods

This prospective observational study included 340 MICU patients (182 survivors and 158 non-survivors). Admission GCS and FOUR scores were recorded. Discrimination was assessed using the area under the receiver operating characteristic (AUROC) curves with 95% confidence intervals (CIs), and optimal cutoffs were determined using Youden's index. Sensitivity, specificity, positive predictive value (PPV), and negative predictive value (NPV) were calculated. Calibration was assessed using Brier score, calibration slope and intercept, and expected calibration error. Multivariable logistic regression analysis was additionally performed to assess the independent association with in-hospital mortality after adjustment for clinically relevant covariates.

Results

The FOUR score demonstrated modestly better discrimination for the prediction of in-hospital mortality compared with GCS, with AUROC curves of 0.799 (95% CI: 0.753-0.844) and 0.731 (95% CI: 0.678-0.783), respectively. The difference in AUROC curves was statistically significant (DeLong p<0.001). Optimal cutoffs were 12.5 for FOUR and 10.5 for GCS. Sensitivity, specificity, PPV, and NPV were 68%, 77%, 72%, and 74% for FOUR and 55%, 84%, 75%, and 68% for GCS, respectively. Calibration assessment using Brier score, calibration slope and intercept, and expected calibration error demonstrated more favorable calibration for the FOUR score. In multivariable logistic regression analysis, both scores remained independently associated with in-hospital mortality after adjustment for age, sex, intubation status, comorbidities, and diagnostic category.

Conclusions

The FOUR score demonstrated modestly better discrimination and more favorable calibration than GCS for the prediction of in-hospital mortality among non-traumatic MICU patients. In addition to improved prognostic performance, the FOUR score offers practical advantages in intubated patients due to the inclusion of brainstem reflexes and respiratory assessment. Further multicenter external validation studies are warranted.

## Introduction

Assessing consciousness is essential in the management of critically ill patients. Consciousness comprises both arousal (wakefulness) and awareness of self and environment, and impairment of these components may occur in conditions such as traumatic brain injury (TBI), metabolic disturbances, infections, poisoning, and systemic illnesses [1-3]. In intensive care units (ICUs), assessment of consciousness plays an important role in neurological monitoring, prognostication, ventilatory decision-making, sedation assessment, and overall clinical management [4,5].

The Glasgow Coma Scale (GCS), introduced in 1974, remains the most widely used tool for evaluating consciousness. It assesses eye, verbal, and motor responses, with scores ranging from 3 to 15, where higher scores indicate better neurological function [6-14]. Its simplicity and ease of bedside application have contributed to widespread adoption across emergency, neurosurgical, and critical care settings. However, the GCS has important limitations, particularly in intubated patients where verbal response cannot be adequately assessed. Furthermore, it does not evaluate brainstem reflexes or respiratory patterns, which may provide additional neurological and prognostic information in critically ill patients [15-17].

To address these limitations, the Full Outline of UnResponsiveness (FOUR) score was developed. In addition to eye and motor responses, the FOUR score incorporates the assessment of brainstem reflexes and respiratory pattern, making it potentially more useful in intubated and critically ill patients [1]. Several studies have compared the FOUR score and GCS in different clinical settings and have suggested that the FOUR score may provide additional prognostic information in selected patient populations [15-25]. However, findings across studies remain variable, and most available studies have focused predominantly on TBI, emergency department populations, or mixed neurological cohorts.

The applicability of these findings to non-traumatic medical intensive care unit (MICU) patients remains uncertain because the causes and pathophysiology of impaired consciousness in this population differ substantially from traumatic neurological conditions. In non-traumatic MICU patients, altered consciousness may result from sepsis, metabolic derangements, respiratory failure, hepatic or renal dysfunction, poisoning, or multisystem illness rather than direct structural brain injury. These patients frequently require intubation, mechanical ventilation, and prolonged critical care, making accurate bedside neurological assessment particularly important for prognostication and risk stratification.

Therefore, this prospective observational study compared the predictive performance of GCS and FOUR scores for in-hospital mortality among non-traumatic patients admitted to the MICU of a tertiary care center. Specifically, the study evaluated discrimination, calibration, optimal cutoff values, and performance across clinically relevant subgroups. This comparison aimed to support early risk stratification and inform the practical selection of prognostic tools in resource-limited ICU settings. We hypothesized that the FOUR score would demonstrate superior predictive performance compared with GCS, particularly in intubated patients, due to the inclusion of brainstem reflexes and respiratory assessment.

## Materials and methods

This prospective observational study was conducted in the MICU of Patan Hospital, Lagankhel, Lalitpur, Nepal, from June 2023 to September 2024. The study compared the predictive performance of the GCS and the FOUR score for predicting in-hospital mortality among non-traumatic patients admitted to the MICU. The study assessed discrimination, optimal cutoff values, sensitivity, specificity, predictive values, correlation, calibration, and adjusted prognostic association of the two scoring systems.

Ethical clearance was obtained from the Institutional Review Committee of Patan Academy of Health Sciences (IRC-PAHS) (approval number: PMM2306301757) before study commencement on June 30, 2023. Written informed consent was obtained either from the patient, when possible, or from the patient's guardian if the patient was unconscious or unable to provide consent.

All data were collected prospectively from MICU admissions. Demographic variables, including age and sex, along with GCS and FOUR scores, were recorded separately for each patient. GCS and FOUR scores were calculated using MDCalc as a bedside checklist/calculator to facilitate the structured application of score components. Individual neurological findings were assessed clinically, and the observed score components were entered into MDCalc to generate the final score. The scoring systems used were based on the original descriptions by Teasdale et al. and Wijdicks et al., respectively [26,27].

The primary investigator personally performed admission-time GCS and FOUR score assessments whenever available. When the primary investigator was unavailable, assisting residents helped with initial patient identification, consent, and preliminary score documentation after an approximately one-hour orientation regarding the scoring systems and data collection process. Formal competency assessment and inter-rater reliability testing were not performed.

Before the completion of the structured proforma and final data entry, all cases underwent verification by the primary investigator to confirm the completeness and reliability of admission-time GCS and FOUR score documentation. Verification required confirmation that both scores were obtained at MICU admission and that sufficient clinical documentation was available to support score components. Cases in which admission-time scoring could not be verified or score components were incomplete were excluded before final analysis. Because the required sample size was achieved using complete and verifiable cases assessed or verified by the primary investigator, the final analysis was restricted to these cases to maintain the internal consistency of neurological scoring.

Subgroup analyses were performed based on age, sex, primary diagnosis, intubation status, comorbidities, and length of hospital stay. During the study period, data were stored in a password-protected computer accessible to the investigator and department. Following study completion, data were deposited in a password-protected departmental/institutional computer for at least five years, with access available to IRC-PAHS/EC-PAHS when required.

The components of the FOUR score and GCS used in this study are shown in Table 1 and Table 2, respectively.

**Table 1:** Components of the Full Outline of UnResponsiveness score Adapted from Wijdicks et al. [26]

Eye opening	Score	Motor response (upper extremities)	Score	Brainstem reflexes	Score	Respiratory pattern	Score
Eyelids open or opened, tracking, or blinking to command	4	Thumbs up, fist or peace sign	4	Pupil and corneal reflexes present	4	Not intubated, regular breathing pattern	4
Eyelids open but not tracking	3	Localizing to pain	3	One pupil wide and fixed	3	Not intubated, Cheyne-Stokes breathing	3
Eyes closed but open to loud voice	2	Flexion response to pain	2	Pupil or corneal reflex absent	2	Not intubated, irregular breathing	2
Eyes closed but open to pain	1	Extension response to pain	1	Pupil and corneal reflexes absent	1	Breathes above ventilator rate	1
Eyes remain closed with pain	0	No response to pain or generalized myoclonus status	0	Absent pupil, corneal, and cough reflexes	0	Breathes at ventilator rate or apnea	0

**Table 2:** Components of the Glasgow Coma Scale Adapted from Teasdale et al. [27]

Best eye response	Score	Best verbal response	Score	Best motor response	Score
Spontaneously	4	Oriented	5	Obeys commands	6
To verbal command	3	Confused	4	Localizes pain	5
To pain	2	Inappropriate words	3	Withdrawal from pain	4
No eye opening	1	Incomprehensible sounds	2	Flexion to pain	3
		No response	1	Extension to pain	2
				No response	1

The sample size was calculated for the comparison of two correlated area under the receiver operating characteristic (AUROC) curves for predicting in-hospital mortality. Expected AUROC curves of 0.75 for the GCS and 0.85 for the FOUR score were derived from previous literature [5]. A 95% confidence level, an 80% power, and an assumed correlation coefficient of 0.5 between the two AUROC curve estimates were used. Although previous studies have generally reported higher correlations between the two scores, a correlation coefficient of 0.5 was selected as a conservative estimate representing the minimum clinically meaningful moderate correlation. The calculated minimum sample size was 156 patients in each outcome group, yielding a total minimum required sample size of 312 patients. The final analyzed sample included 340 patients, exceeding the calculated minimum requirement. Detailed sample size calculations are provided in the Appendices.

All statistical analyses were performed using R Version 4.4.1 (R Foundation for Statistical Computing, Vienna, Austria). Discrimination was assessed using AUROC curves with 95% confidence intervals (CIs), and AUROC curves of GCS and FOUR scores were compared using DeLong's test. Optimal cutoff values were determined using Youden's index. Sensitivity, specificity, positive predictive value (PPV), and negative predictive value (NPV) were calculated. Correlation between GCS and FOUR scores was assessed using Spearman's correlation analysis. Calibration was assessed using Brier score, calibration slope and intercept, and expected calibration error.

Multivariable logistic regression analysis was additionally performed to assess the independent association of GCS and FOUR scores with in-hospital mortality after adjustment for clinically relevant covariates including age, sex, intubation status, comorbidities, and diagnostic category. Separate regression models were constructed for GCS and FOUR scores to avoid multicollinearity due to the strong correlation between the two neurological scoring systems. Adjusted odds ratios (ORs) with 95% CIs were calculated. Model fit was assessed using the Akaike Information Criterion (AIC) and McFadden pseudo-R². Multicollinearity was assessed using variance inflation factors (VIFs). A p-value of less than 0.05 was considered statistically significant.

## Results

**Figure 1:**
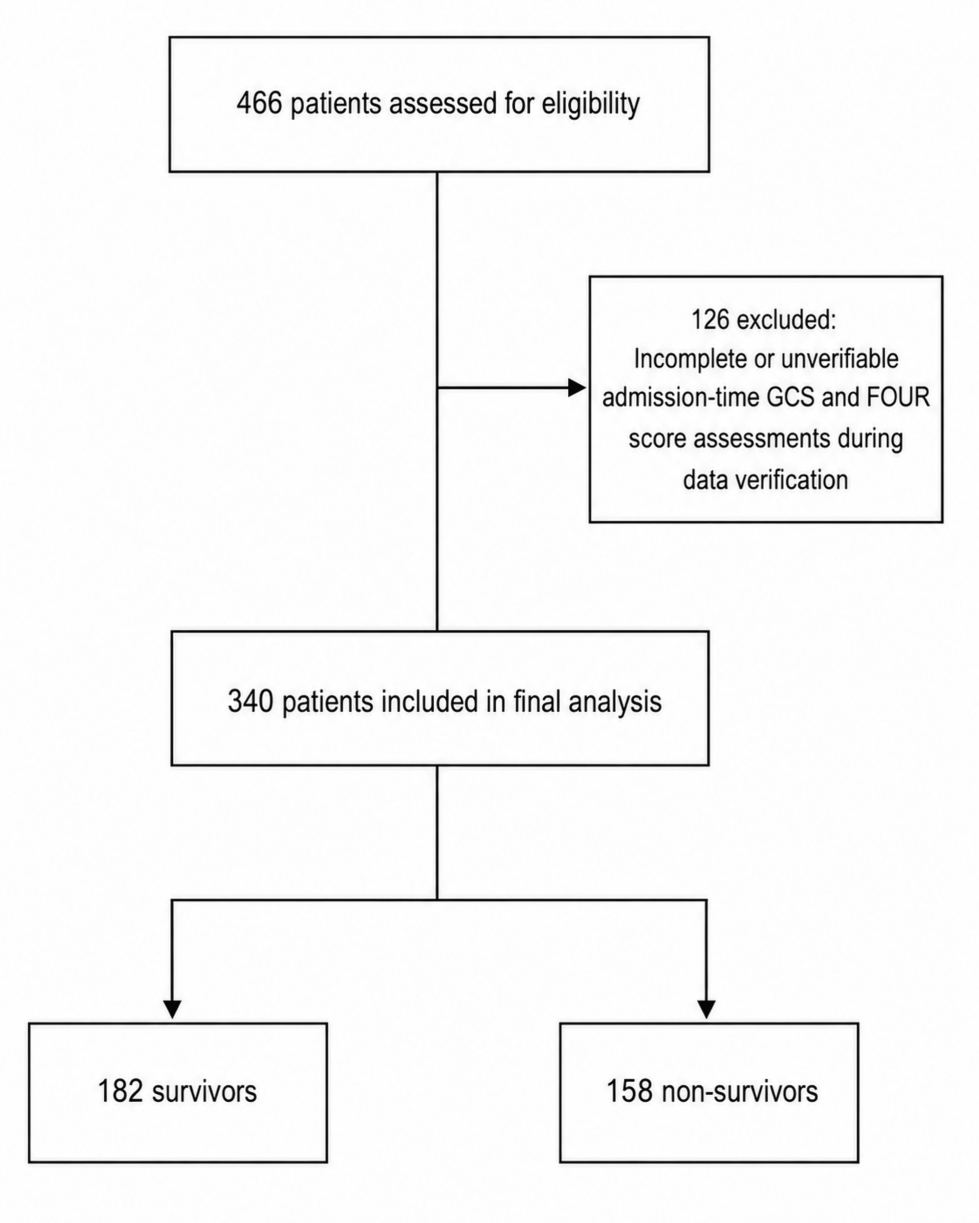
Flowchart of study selection GCS: Glasgow Coma Scale; FOUR: Full Outline of UnResponsiveness

A total of 466 patients were assessed for eligibility. During data verification, 126 patients were excluded because complete and verifiable admission-time GCS and FOUR score assessments were unavailable. Therefore, 340 patients were included in the final analysis. Among the analyzed patients, 182 survived, and 158 died during MICU admission (Figure 1).

Age

The age range of the patients was 18-98 years, with a mean age of 54.97±20.57. The mean age in survivors was 51.26±21.246 years, while the mean age in non-survivors was 59.19±19.075. In the age categories <40 years, 40-60 years, and >60 years, the number of patients admitted was 93 (27.35%), 91 (26.76%), and 156 (45.89%), respectively. The p-value obtained was 0.012, suggesting a significant difference as per age and outcome. Age played a significant role in determining survival outcomes, with patients aged <40 years exhibiting the highest survival odds (OR: 2.105; 95% CI: 1.235-3.589; p=0.012) compared to the reference group aged >60 years. Patients aged 40-60 years showed similar survival outcomes to the ">60 years" group (OR: 0.989; 95% CI: 0.590-1.660), highlighting a potential threshold effect of younger age on better prognosis.

Sex

The male population was 167 (49.1%), while the female population was 173 (50.9%). The ratio of male to female was 0.97:1. Sex differences in survival were not statistically significant, although female patients showed slightly higher odds of survival compared to males (OR: 1.354; 95% CI: 0.883-2.077; p=0.164).

Length of stay

The mean length of hospital stay was 10.67±8.973 days. The mean length of stay in survivors was 11.78±8.876 days, whereas the mean length of hospital stay in non-survivors was 9.38±8.976 days. The p-value was calculated to be 0.004, which is significant. Thus, there is a statistically significant association between stay categories and outcomes, i.e., length of hospital stay also influenced survival. Patients with intermediate stays of 10-30 days had significantly better survival odds (OR: 2.153; 95% CI: 1.366-3.393; p=0.004) compared to those staying <10 days. However, stays exceeding 30 days did not confer additional survival benefits (OR: 1.341; 95% CI: 0.468-3.846). 

Systemic involvement

The respiratory system was involved the most with 95 patients (27.9%). Hematology and poisoning constitute the least number of patients, i.e., 13 in number (3.8%). The primary diagnosis significantly influenced survival outcomes, as evidenced by a p-value of 0.001 for the overall comparison across all diagnostic groups. Among the diagnoses, metabolic disorders demonstrated the highest odds of survival (OR: 4.787; 95% CI: 1.507-15.206), which was statistically significant. Similarly, poisoning cases showed significantly better survival odds compared to respiratory conditions (OR: 3.543; 95% CI: 1.307-9.602). This may reflect the reversibility of underlying conditions and response to timely supportive management. On the other hand, hematological conditions were significantly associated with low survival odds (OR: 0.191; 95% CI: 0.040-0.911). In the hematological group, a smaller sample size may limit the power to detect association, necessitating cautious interpretation. Other diagnostic groups, such as neurology (OR: 1.468; 95% CI: 0.687-3.137), cardiovascular (OR: 1.383; 95% CI: 0.553-3.461), nephrology (OR: 0.617; 95% CI: 0.224-1.703), gastroenterology (OR: 1.106; 95% CI: 0.560-2.185), and infectious diseases (OR: 0.872; 95% CI: 0.416-1.831) and monitoring (OR: 3.543; 95% CI: 0.917-13.686) did not show statistically significant differences in survival odds compared to respiratory conditions.

Status of endotracheal intubation

Eighty-four patients (24.7%) underwent endotracheal intubation, whereas 256 patients (75.3%) were not intubated during their admission to MICU. The survival odds of the intubated category were 0.217. In contrast, non-intubated patients had a markedly higher survival rate, with survival odds of 1.876. The odds ratio for non-intubated patients was 8.645 (95% CI: 4.675-15.987), highlighting a statistically significant survival advantage compared to intubated patients.

Comorbidities

A total of 107 patients (31.47%) had no comorbidities. The rest of the 233 patients (68.53%) had various comorbidities. The most common comorbidity was hypertension (20.88%), followed by diabetes mellitus (15.59%). The presence of comorbidities was a significant factor influencing survival, with a p-value of 0.001, indicating a strong association with outcomes. Patients with comorbidities (n=233) had a lower survival rate, with 110 survivors (47.21%) and 123 non-survivors (52.79%). This resulted in survival odds of 0.894, which served as the reference group. In contrast, patients without comorbidities (n=107) showed a significantly higher survival rate, with 72 survivors (67.29%) and 35 non-survivors (32.71%), and survival odds of 2.057. The odds ratio for patients without comorbidities was 2.3 (95% CI: 1.425-3.714), highlighting a statistically significant survival advantage compared to those with comorbidities. These findings suggest that the absence of comorbidities confers a notable survival benefit, likely due to the greater physiological reserve and reduced baseline disease burden in these patients.

GCS score

The minimum GCS score was 3, while the maximum was 15. The mean GCS was 10.96±4.752. The mean GCS score in survivors was 12.88±3.378, whereas the mean GCS in non-survivors was 8.75±5.141. The Kolmogorov-Smirnov test showed that the distribution of GCS scores was non-normal, with a median of 14 and an interquartile range of 6-15 overall. The median GCS scores with interquartile range in survivors and non-survivors were 15 (12-15) and 8 (3-14), respectively. The Mann-Whitney U test suggested a significant difference in the distribution of GCS scores among survivors and non-survivors (p<0.001). 

FOUR score

The minimum FOUR score was 1, while the maximum was 16. The mean FOUR score was 11.6±4.471. The mean FOUR score in survivors was 13.71± 2.888, whereas the mean FOUR score in non-survivors was 9.16±4.729. The Kolmogorov-Smirnov test showed that the distribution of FOUR scores was non-normal, with a median of 13 and an interquartile range of 8.25-15 overall. The median FOUR scores with interquartile range in survivors and non-survivors were 14 (13-16) and 11 (5-14), respectively. The Mann-Whitney U test suggested a significant difference in the distribution of FOUR scores among survivors and non-survivors (p<0.001).

Table 3 presents a summary of patient characteristics.

**Table 3:** Summary of patient characteristics *The number of patients in GCS high/GCS low and FOUR high/FOUR low has been decided based on the best cutoff (see Table 4: any value more than the cutoff was categorized as high; a value less than the cutoff was classified as low) GCS: Glasgow Coma Scale; FOUR: Full Outline of UnResponsiveness

Characteristics	Total (N)	Survivors (n, %)	Non-survivors (n, %)	Odds of survival	Odds ratio (95% CI)	χ² statistic	P-value
Age (years)							
<40	93	62 (66.67)	31 (33.33)	2	2.105 (1.235-3.589)	8.885	0.012
40-60	91	44 (48.35)	47 (51.65)	0.94	0.989 (0.590-1.660)		
>60	156	76 (48.7)	80 (51.3)	0.95	1 (reference)		
Sex							
Male	167	83 (49.7)	84 (50.3)	0.988	1 (reference)	1.934	0.164
Female	173	99 (57.23)	74 (42.77)	1.338	1.354 (0.883-2.077)		
Stay							
<10 days	192	88 (45.83)	104 (54.17)	0.85	1 (reference)	11.198	0.004
10-30 days	133	86 (64.66)	47 (35.34)	1.83	2.153 (1.366-3.393)		
>30 days	15	8 (53.33)	7 (46.67)	1.14	1.341 (0.468-3.846)		
GCS							
High*	224	153 (68.3)	71 (31.7)	2.155	6.471 (3.902-10.732)	57.611	0.001
Low*	116	29 (25)	87 (75)	0.333	1 (reference)		
Median (IQR)	14 (6-15)	15 (12-15)	8 (3-14)	-	-	-	-
FOUR							
High*	190	140 (73.7)	50 (26.3)	2.8	7.198 (4.450-11.643)	70.328	<0.001
Low*	150	42 (28)	108 (72)	0.389	1 (reference)		
Median(IQR)	13 (8.25-15)	14 (13-16)	11 (5-14)	-	-	-	-
Diagnosis							
Neurology	38	22 (57.89)	16 (42.11)	1.38	1.468 (0.687-3.137)	28.062	0.001
Respiratory	95	46 (48.42)	49 (51.58)	0.94	1 (reference)		
Cardiovascular	23	13 (56.52)	10 (43.48)	1.3	1.383 (0.553-3.461)		
Nephrology	19	7 (36.84)	12 (63.16)	0.58	0.617 (0.224-1.703)		
Gastroenterology	51	26 (50.98)	25 (49.02)	1.04	1.106 (0.560-2.185)		
Infectious	40	18 (45)	22 (55)	0.82	0.872 (0.416-1.831)		
Metabolic	22	18 (81.82)	4 (18.18)	4.5	4.787 (1.507-15.206)		
Hematology	13	2 (15.38)	11 (84.62)	0.18	0.191 (0.040-0.911)		
Poisoning	26	20 (76.92)	6 (23.08)	3.33	3.543 (1.307-9.602)		
Monitoring	13	10 (76.92)	3 (23.08)	3.33	3.543 (0.917-13.686)		
Comorbidities							
Present	233	110 (47.21)	123 (52.79)	0.894	1 (reference)	11.885	0.001
Absent	107	72 (67.29)	35 (32.71)	2.057	2.3 (1.425-3.714)		
Status of intubation							
Yes	84	15 (17.86)	69 (82.14)	0.217	1 (reference)	57.070	<0.001
No	256	167 (65.23)	89 (34.77)	1.876	8.645 (4.675-15.987)		

ROC curve analysis

**Figure 2:**
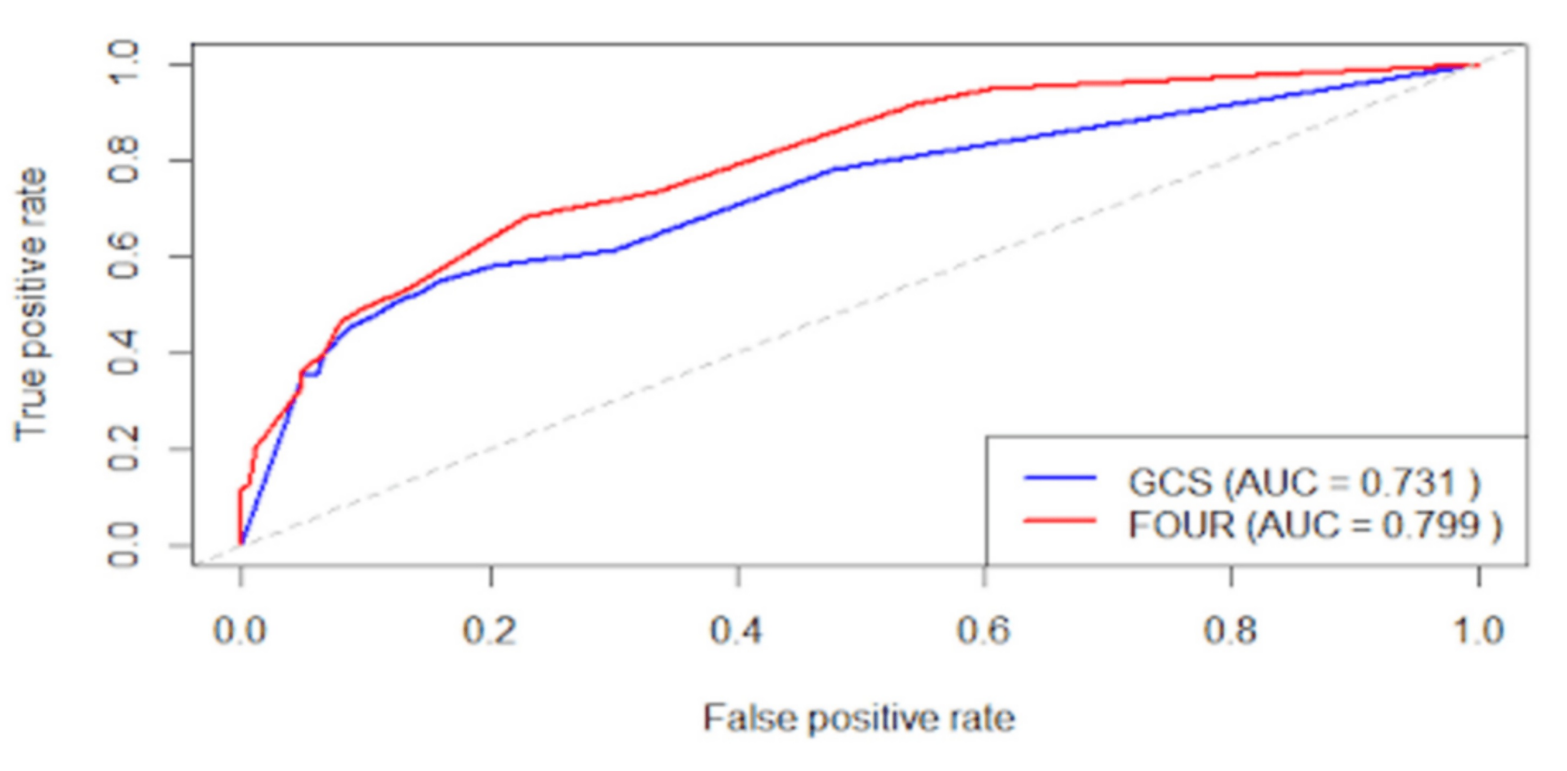
ROC curves for GCS and FOUR scores ROC: receiver operating characteristic; GCS: Glasgow Coma Scale; FOUR: Full Outline of UnResponsiveness

ROC curve analysis was performed to evaluate the ability of GCS and FOUR scores to predict in-hospital mortality. The AUROC curve for GCS was 0.731 (95% CI: 0.678-0.783), indicating acceptable discriminatory ability. The AUROC curve for FOUR was 0.799 (95% CI: 0.753-0.844), demonstrating modestly better discrimination than GCS. Comparison of the two AUROC curves using DeLong's test showed that this difference was statistically significant (p<0.001) (Figure 2). 

Best cutoff and Youden's index

Youden's index was used to determine the optimal cutoff value [28] (Table 4).

**Table 4:** Best cutoff for GCS and FOUR with corresponding Youden's index GCS: Glasgow Coma Scale; FOUR: Full Outline of UnResponsiveness

Characteristics	Youden's index	Best cutoff
GCS	0.392	10.5
FOUR	0.453	12.5

Correlation between GCS and FOUR scores

A significant correlation (p<0.001) with Spearman's rho of 0.871 was seen between GCS and FOUR scores.

Calibration

For GCS, the calibration intercept was 0.185 (95% CI: 0.051-0.319).

The positive calibration intercept of 0.185 suggests that, on average, the model underestimates the likelihood of the outcome, particularly at the lower end of the predicted probabilities.

Further, an ideally calibrated model would have an intercept close to 0, indicating no systematic bias in predictions across all levels of risk. The 95% CI for the intercept, ranging from 0.051 to 0.319, does not include 0, which means that this underestimation is statistically significant.

For GCS, the calibration slope was 0.528 (95% CI: 0.355-0.702).

A calibration slope of 0.528 suggests that the model's predictions are too aggressive compared to the observed outcomes. In practical terms, this means that the GCS model does not adequately differentiate between high-risk and low-risk cases. The 95% CI for the slope (0.355-0.702) does not include 1, confirming that the model deviates significantly from perfect calibration.

For GCS, the expected calibration error (ECE) was 0.204 (95% CI: 0.131-0.280). 

**Figure 3:**
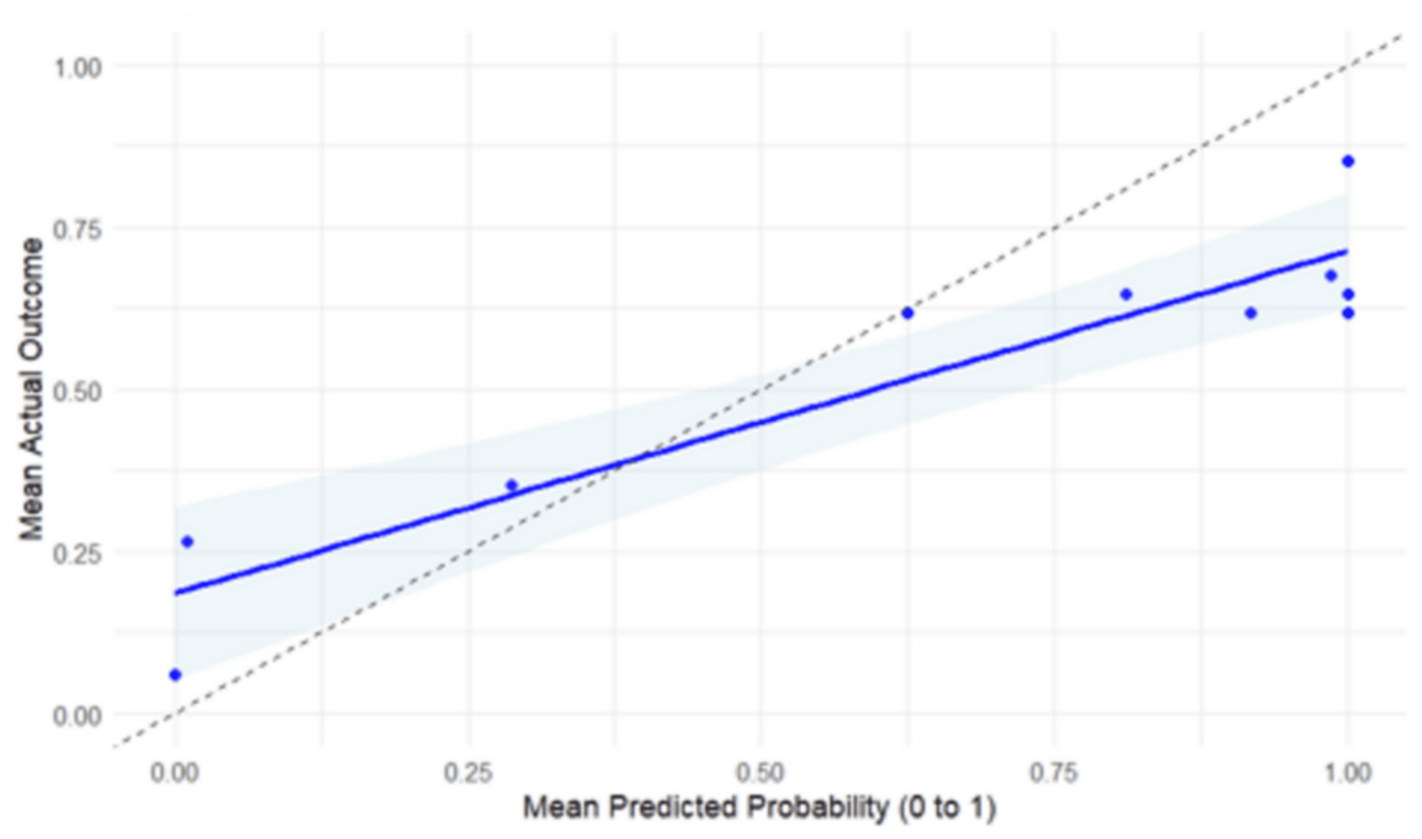
Calibration plot for GCS score GCS: Glasgow Coma Scale

The ECE of 0.204 represents the average absolute difference between predicted probabilities and observed outcomes across bins. A lower ECE indicates better calibration. The 95% CI for ECE (0.131-0.280) provides a range within which the true average calibration error likely lies (Figure 3).

For FOUR, the calibration intercept was -0.122 (95% CI: -0.347 to 0.102). 

The slightly negative intercept of -0.122 suggests that the model overestimates the likelihood of the outcome at lower predicted probability levels. An ideally calibrated model would have an intercept close to 0, indicating no systematic bias across the risk spectrum.

Since the 95% CI for the intercept (-0.347 to 0.102) includes 0, the intercept is not statistically significantly different from 0, indicating minimal bias in predictions across all levels of risk.

For FOUR, the calibration slope was 0.907 (95% CI: 0.617-1.197).

A calibration slope of 0.907, close to 1, suggests that the model's predictions are relatively well-calibrated. This indicates that the FOUR model does a reasonable job of differentiating between high-risk and low-risk cases.

Since the 95% CI for the slope (0.617-1.197) includes 1, the model is not statistically significantly different from perfect calibration, meaning it aligns well with the observed outcomes across the risk spectrum.

For FOUR, the ECE was 0.190 (95% CI: 0.133-0.254).

**Figure 4:**
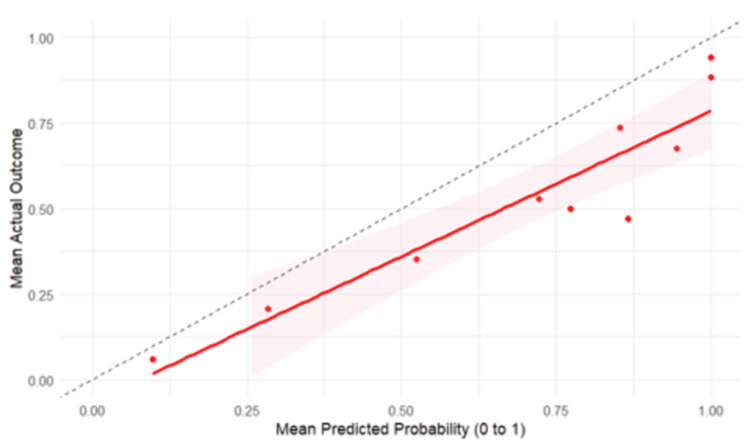
Calibration plot for FOUR score FOUR: Full Outline of UnResponsiveness

The ECE of 0.190 represents the average absolute difference between the predicted probabilities and observed outcomes across bins. Lower ECE values indicate better calibration (Figure 4).

Table 5 shows the calibration analysis of GCS and FOUR scores.

**Table 5:** Calibration analysis of GCS and FOUR scores GCS: Glasgow Coma Scale; FOUR: Full Outline of UnResponsiveness

Metrics	GCS model	FOUR model
Intercept	0.185 (95% CI: 0.051 to 0.319)	-0.122 (95% CI: -0.347 to 0.102)
Slope	0.528 (95% CI: 0.355 to 0.702)	0.907 (95% CI: 0.617 to 1.197)
Expected calibration error	0.204 (95% CI: 0.131 to 0.280)	0.190 (95% CI: 0.133 to 0.254)

Brier score and Brier decomposition

The accuracy of predicted probabilities was evaluated using the Brier score and its decomposition [29]. For the GCS model, the Brier score was calculated to be 0.251. The reliability measure was 0.054. Resolution was estimated to be 0.049. The uncertainty measure was found to be 0.249. Similarly, for the FOUR model, the Brier score was calculated to be 0.221. Reliability and resolution measures were calculated to be 0.046 and 0.071, respectively. The uncertainty measure was the same as the GCS model, i.e., 0.249.

The FOUR model has a lower Brier score than the GCS model (0.221 vs. 0.251), suggesting it provides better overall accuracy. Calibration (reliability) is also better in the FOUR model, as indicated by the lower reliability measure (0.046 vs. 0.054), meaning that the FOUR model's predicted probabilities are closer to actual outcomes. The obtained reliability measures confirm the inference obtained from calibration metrics, which suggest that the FOUR score is better calibrated than GCS. Resolution is higher in the FOUR model (0.071 vs. 0.049), showing that it has a better discriminative power. Uncertainty is the same for both models and reflects the underlying variability of the outcome in the dataset, not the model performance itself (Table 6).

**Table 6:** Brier score and its decomposition between GCS and FOUR GCS: Glasgow Coma Scale; FOUR: Full Outline of UnResponsiveness

Characteristics	GCS	FOUR
Brier score	0.251	0.221
Uncertainty	0.249	0.249
Reliability	0.054	0.046
Resolution	0.049	0.071

Subgroup analysis

Sensitivity, specificity, PPV, and NPV were calculated with mortality as the outcome unless otherwise stated.

Gender-specific analysis revealed that the FOUR score consistently outperformed the GCS in both male and female patients. Among males, the FOUR score achieved an AUROC curve of 0.813 compared to 0.738 for the GCS, with improved sensitivity (0.68 vs. 0.55) and NPV (0.72 vs. 0.65). Similarly, in females, the FOUR score demonstrated an AUROC curve of 0.789 compared to 0.723 for the GCS, with a slightly better PPV (0.75 vs. 0.72). The correlation between the scores was slightly higher in females (rho=0.873) than in males (rho=0.870).

In patients requiring intubation, the FOUR score showed marked improvements over the GCS, achieving an AUROC curve of 0.721 versus 0.648 for the GCS, reflecting a 7.3% improvement. The specificity of the FOUR score in this subgroup was significantly higher (0.87 vs. 0.40 for GCS), indicating a better ability to identify survivors. However, this came at the expense of sensitivity, which was higher for the GCS (0.93 vs. 0.46 for FOUR). In non-intubated patients, the FOUR score also performed better, with an AUROC curve of 0.711 compared to 0.596 for the GCS. The FOUR score demonstrated substantially higher sensitivity (0.85 vs. 0.61) and NPV (0.86 vs. 0.73) in this subgroup, reducing false negatives and enhancing its reliability in identifying non-survivors.

Age-stratified analysis further emphasized the FOUR score's superior discriminative ability. In patients under 40 years, the FOUR score achieved an AUROC curve of 0.830, outperforming the GCS (AUROC curve=0.763) by 6.7%. It also maintained higher sensitivity (0.61 vs. 0.58) and better predictive values. Similar trends were observed in patients aged 40-60 years, where the FOUR score showed an AUROC curve of 0.824 compared to 0.770 for the GCS, with improved sensitivity (0.77 vs. 0.53). Even in patients over 60 years, the FOUR score outperformed the GCS with an AUROC curve of 0.750 compared to 0.682, offering higher sensitivity (0.68 vs. 0.53) for this older population.

System-specific analyses revealed remarkable performance differences. In the hematologic subgroup, the FOUR score showed a higher AUROC curve than GCS (1.000 vs. 0.955). However, this finding should be interpreted cautiously because the hematologic subgroup included only 13 patients. The apparently perfect discrimination may reflect the small sample size and may be prone to overfitting and statistical instability. Similarly, in metabolic conditions, the FOUR score had an AUROC curve of 0.868 versus 0.903 for the GCS, demonstrating its utility in predicting outcomes in diverse clinical contexts. However, both scores performed poorly in poisoning cases, with AUROC curves of 0.658 for the FOUR score and 0.500 for the GCS. Among respiratory cases, the FOUR score showed notable improvements in sensitivity (0.98 vs. 0.51 for GCS) despite lower specificity (Table 7).

**Table 7:** Subgroup analysis AUROC: area under the receiver operating characteristic; GCS: Glasgow Coma Scale; FOUR: Full Outline of UnResponsiveness; Sp: specificity; Sens: sensitivity; NPV: negative predictive value; PPV: positive predictive value

Characteristics	AUROC curve (GCS/FOUR)	Cutoff (GCS/FOUR)	Sp/Sens/NPV/PPV (GCS) for in-hospital mortality	Sp/Sens/NPV/PPV (FOUR) for in-hospital mortality	Spearman's rho
Overall	0.731/0.799	10.5/12.5	0.84/0.55/0.68/0.75	0.77/0.68/0.74/0.72	0.871
Male	0.738/0.813	9.5/12.5	0.86/0.55/0.65/0.79	0.83/0.68/0.72/0.80	0.870
Female	0.723/0.789	10.5/11.5	0.84/0.55/0.72/0.72	0.86/0.55/0.72/0.75	0.873
Intubated	0.648/0.721	6.5/4.5	0.4/0.93/0.55/0.88	0.87/0.46/0.26/0.94	0.701
Not intubated	0.596/0.711	14.5/14.5	0.57/0.61/0.73/0.43	0.49/0.85/0.86/0.47	0.737
Age <40 years	0.763/0.830	7.5/10.5	0.92/0.58/0.81/0.78	0.94/0.61/0.83/0.83	0.855
Age 40-60 years	0.770/0.824	8.5/13.5	0.91/0.53/0.65/0.86	0.73/0.77/0.74/0.75	0.890
Age >60 years	0.682/0.750	10.5/12.5	0.82/0.53/0.62/0.75	0.70/0.68/0.67/0.70	0.859
Stay <10 days	0.779/0.841	11.5/12.5	0.92/0.58/0.65/0.90	0.81/0.72/0.71/0.82	0.879
Stay 10-30 days	0.666/0.730	6.5/9.5	0.87/0.47/0.75/0.67	0.88/0.49/0.76/0.70	0.871
Stay >30 days	0.750/0.848	10.5/15.5	0.75/0.71/0.75/0.71	0.5/1/1/0.64	0.855
Neurology	0.73/0.776	6.5/10.5	0.96/0.56/0.75/0.9	0.96/0.63/0.78/0.91	0.875
Respiratory	0.635/0.743	8.5/14.5	0.78/0.51/0.6/0.71	0.41/0.98/0.95/0.64	0.874
Cardiovascular	0.804/0.815	5.5/6.5	1/0.6/0.76/1	1/0.5/0.72/1	0.915
Nephrology	0.881/0.821	13.5/11.5	0.86/0.83/0.75/0.91	1/0.75/0.7/1	0.933
Gastroenterology	0.789/0.842	14.5/13.5	0.69/0.8/0.78/0.71	0.81/0.76/0.78/0.79	0.863
Infectious	0.739/0.824	11.5/12.5	0.94/0.55/0.63/0.92	0.89/0.64/0.67/0.88	0.927
Metabolic	0.903/0.868	13.5/13.5	0.83/1/1/0.57	0.72/1/1/0.44	0.896
Hematologic	0.955/1	14.5/15.5	1/0.91/0.67/1	1/1/1/1	0.905
Poisoning	0.5/0.658	7.5/14.5	0.9/0.33/0.82/0.5	0.5/0.83/0.91/0.33	0.762
Monitoring	0.95/0.983	13.5/11.5	0.9/1/1/0.75	0.9/1/1/0.75	0.880
Hypertension	0.721/0.795	10.5/12.5	0.91/0.5/0.61/0.86	0.76/0.76/0.74/0.78	0.823

Multivariable logistic regression analysis

On multivariable logistic regression analysis, both GCS and FOUR scores remained independently associated with in-hospital mortality after adjustment for age, sex, intubation status, comorbidities, and diagnostic category. In the GCS model, each one-point increase in GCS was associated with reduced adjusted odds of mortality (aOR: 0.85; 95% CI: 0.77-0.93; p<0.001). In the FOUR score model, each one-point increase in FOUR score was similarly associated with reduced adjusted odds of mortality (aOR: 0.72; 95% CI: 0.64-0.80; p<0.001). The FOUR score model showed better overall model fit than the GCS model, with lower AIC (361.08 vs. 388.02) and higher McFadden pseudo-R² (0.295 vs. 0.238) (Table 8).

**Table 8:** Multivariable logistic regression models for in-hospital mortality GCS: Glasgow Coma Scale; FOUR: Full Outline of UnResponsiveness

Variable	GCS model OR (95% CI), p-value	FOUR model OR (95% CI), p-value
Score	0.85 (0.77-0.93), <0.001	0.72 (0.64-0.80), <0.001
Age	1.01 (1.00-1.03), 0.087	1.01 (1.00-1.03), 0.119
Female sex	0.89 (0.53-1.50), 0.659	0.81 (0.47-1.40), 0.452
Intubated	2.15 (0.76-6.15), 0.147	0.87 (0.30-2.43), 0.794
Comorbidity present	2.57 (1.36-4.98), 0.004	2.96 (1.51-6.00), 0.002

## Discussion

Age

The minimum age in our study was 18 years, and the maximum was 98 years, with a mean of 54.97±20.57. In comparison, Kafle et al. reported an age range of 16-82 years, while Hosseini et al. found a mean age of 63.36±16.98 years [12,15].

Sex

Our study included 49.1% males and 50.9% females (n=340). Many studies report a male predominance, particularly in TBI cases. Keerthi et al. found 72.8% males among TBI patients, while Javvaji et al. reported 59% males in a critically ill ICU population [2,5].

Length of hospital stay

The mean hospital stay was 10.67±8.97 days. Survivors had a mean stay of 11.78±8.87 days, whereas non-survivors had a mean stay of 9.38±8.97 days. Patients staying for 10-30 days had the best survival odds (OR=2.153; CI: 1.366-3.393). Hosseini et al. found an ICU stay mean of 19.02±12.30 days [15].

Correlation between GCS and FOUR scores

This study compared the predictive utility of the GCS and FOUR scores in MICU patients. We observed a strong correlation (Spearman's rho=0.871; p<0.001). In intubated patients, rho was 0.701 (p<0.001), while in non-intubated patients, rho was 0.737 (p<0.001). These findings align with studies by Bruno et al. (rho=0.81) and Kafle et al. (rho=0.92) [11,12].

Discriminative ability of GCS and FOUR scores

The AUROC curve for predicting in-hospital mortality was 0.731 (0.676-0.785) for GCS and 0.799 (0.752-0.845) for FOUR, with p<0.001 for both. This suggests FOUR has better discrimination. In intubated patients, the AUROC curve for GCS was 0.648 (0.474-0.821; p=0.074), while for FOUR, it was 0.721 (0.586-0.855; p=0.008). In non-intubated patients, the AUROC curve for GCS was 0.596 (0.522-0.669; p=0.012), whereas for FOUR, it was 0.711 (0.647-0.776; p<0.001).

Comparing these with prior studies, Kafle et al. reported AUROC curve values of 0.975 for GCS and 0.981 for FOUR (p<0.001) [12]. Okasha et al. reported AUROC curve values of 0.85 for FOUR and 0.796 for GCS (p=0.025) [21]. Khanal et al. found AUROC curve values of 0.79 (GCS) and 0.82 (FOUR) [23]. Eken et al. reported AUROC curve values of 0.735 (GCS) and 0.788 (FOUR) [24].

Best cutoff values

The best cutoffs for predicting in-hospital mortality in our study were 10.5 for GCS and 12.5 for FOUR, with Youden indices of 0.392 and 0.453, respectively. For intubated patients, the optimal cutoffs were 6.5 (GCS) and 4.5 (FOUR). Kafle et al. found GCS scores <7 and FOUR scores <8 associated with high mortality [12]. Khanal et al. identified a cutoff of 6.5 for both scores, while Eken et al. determined 5 for GCS and 9 for FOUR [23,24]. These differences in cutoffs may reflect variation in clinical setting and patient population, as emergency department and trauma studies often include patients assessed earlier after acute injury, whereas non-traumatic MICU patients may have altered consciousness due to metabolic, infectious, respiratory, toxicological, or multisystem illness and may already have received ICU interventions such as ventilation or sedation.

Sensitivity, specificity, and predictive values

For overall mortality prediction, sensitivity, specificity, PPV, and NPV were 55%/84%/75%/68% for GCS and 68%/77%/72%/74% for FOUR. In intubated patients, the GCS model had sensitivity/specificity/PPV/NPV of 93%/40%/88%/55%, while FOUR had 46%/87%/94%/26%. In non-intubated patients, these values for GCS were 61%/57%/43%/73%, while for FOUR, they were 85%/49%/47%/86%. These findings suggest that the two scores may provide complementary bedside information. GCS may remain useful because of its simplicity, familiarity, and relatively high sensitivity in intubated patients, whereas FOUR may add value by incorporating brainstem reflexes and respiratory pattern, improving specificity in intubated patients and sensitivity in non-intubated patients. Therefore, using both scores together may help clinicians identify high-risk MICU patients more comprehensively than relying on either score alone.

Comparing neurological patients in our study, GCS had specificity/sensitivity/NPV/PPV of 96%/56%/75%/90%, while FOUR had 96%/63%/78%/91%. Jalali et al. found sensitivity, specificity, and PPV of 68%/63%/52% for GCS and 68%/77%/63% for FOUR [10]. Kafle et al. reported 91.67%/91.82%/57.1%/100% for GCS and 100%/91.82%/55%/99% for FOUR [12]. Akavipat et al. found GCS superior in sensitivity, specificity, and PPV, while FOUR had higher NPV [25].

Calibration analysis

The findings regarding calibration are consistent with research by Furman et al. and Khanal et al., which highlighted the FOUR score's superior calibration over the GCS in ICU settings [4,23]. In contrast, Agrawal et al. suggested that calibration power was good for GCS only [7].

Multivariable analysis

Multivariable logistic regression was performed to assess whether GCS and FOUR scores remained independently associated with in-hospital mortality after adjustment for clinically relevant covariates. Both scores remained significant independent predictors of mortality after adjustment for age, sex, intubation status, comorbidities, and diagnostic category. Each one-point increase in GCS was associated with lower adjusted odds of mortality, and each one-point increase in FOUR showed a similar protective association. This supports the independent prognostic value of both neurological scoring systems in non-traumatic MICU patients. Because this was an observational study, these adjusted findings should be interpreted as independent associations rather than causal effects.

Limitations of the study

This study has several limitations. First, 126 initially assessed patients were excluded because complete and verifiable admission-time GCS and FOUR score assessments were unavailable according to the predefined methodology. Some preliminary assessments were documented by assisting residents when the primary investigator was unavailable; however, cases were excluded when the timing of admission assessment or score components could not be verified. Because detailed patient-level data were not extracted for excluded cases, comparison between included and excluded patients could not be performed. Therefore, the possibility of selection bias related to these exclusions cannot be completely excluded.

Second, although assisting residents received an approximately one-hour orientation regarding the scoring systems and data collection process, formal competency assessment and inter-rater reliability testing were not performed. Restricting the final analysis to complete and verifiable cases assessed or verified by the primary investigator improved the internal consistency of neurological scoring, but observer-related bias cannot be completely excluded.

Third, this was a single-center study conducted in the MICU of one tertiary hospital, which may limit generalizability to other hospitals, ICU populations, and healthcare settings. Fourth, external validation was not performed; therefore, the predictive performance of GCS and FOUR scores should be evaluated in larger multicenter cohorts before broad application.

Fifth, critically ill MICU patients often have overlapping diagnoses, comorbidities, treatment exposures, and multisystem involvement, which may confound the relationship between neurological scores and outcomes. Although multivariable logistic regression was performed to adjust for clinically relevant covariates, residual confounding cannot be excluded. Finally, findings from smaller diagnostic subgroups, such as the hematology group, should be interpreted cautiously because small subgroup sizes may produce unstable estimates.

## Conclusions

In this prospective observational study of non-traumatic MICU patients, both GCS and FOUR scores were useful for predicting in-hospital mortality. The FOUR score demonstrated modestly better discrimination and more favorable calibration than GCS. Although the absolute AUROC curve difference was moderate, calibration metrics more clearly favored the FOUR score, with a calibration intercept closer to 0, a calibration slope closer to 1, a lower expected calibration error, and a lower Brier score. On multivariable logistic regression analysis, both scores remained independently associated with in-hospital mortality after adjustment for clinically relevant covariates, while the FOUR-based model demonstrated better overall model fit.

These findings suggest that the FOUR score may provide additional prognostic information beyond GCS, particularly because it incorporates brainstem reflexes and respiratory pattern assessment, which are clinically relevant in intubated and critically ill patients. However, this was a single-center study without external validation, and the outcome was limited to in-hospital mortality without assessment of functional outcomes or long-term follow-up. Larger multicenter studies are warranted to validate the prognostic performance and clinical applicability of the FOUR score in non-traumatic MICU populations.

## Appendices

Calculation of sample size

The sample size was calculated as follows: the number of cases needed to detect an effect between two diagnostic tasks as defined by δ = AUC1 − AUC2 under the alternative hypothesis with (1 − α)% confidence level and (1 − β)% power. By constructing a confidence interval for the parameter of interest, AUC1 − AUC2, using normal approximation under the null and alternative hypotheses, the required sample size is given by the formula below [30]:

\[ n = \frac{ \left[ Z_{\alpha/2} \sqrt{V_{H0}(\widehat{AUC}_1 - \widehat{AUC}_2)} + Z_{\beta} \sqrt{V_{H1}(\widehat{AUC}_1 - \widehat{AUC}_2)} \right]^2 }{ (\widehat{AUC}_1 - \widehat{AUC}_2)^2 } \]

where

\begin{align*} V(\widehat{AUC}_1 - \widehat{AUC}_2) &= n\,\mathrm{Var}(\widehat{AUC}_1) + n\,\mathrm{Var}(\widehat{AUC}_2) - 2n\,\mathrm{Cov}(\widehat{AUC}_1, \widehat{AUC}_2) \\ n\,\mathrm{Var}(\widehat{AUC}) &= V(AUC) = (0.0099 \times e^{-a^2/2}) \times (6a^2 + 16) \\ a &= \phi^{-1}(\mathrm{AUC}) \times 1.414 \\ \mathrm{Cov}(\widehat{AUC}_1, \widehat{AUC}_2) &= r\,\mathrm{SE}(\widehat{AUC}_1) \cdot \mathrm{SE}(\widehat{AUC}_2) \end{align*}

Z α/2 and Z β denote the upper α/2 and β percentiles of the standard normal distribution, and 

α and β are the probabilities of type I and type II errors, respectively.

Z α/2=1.96 and Z β=0.84 for α=0.05 and β=0.20.

H0:null hypothesis: AUC1=AUC2; H1: alternate hypothesis:AUC1 ≠ AUC2

AUC1=area under the receiver-operator curve of test 1; here, test 1 is GCS. 

AUC2=area under the receiver-operator curve of test 2; here, test 2 is FOUR.

r=correlation coefficient, SE=standard error=square root of variance, a=φ−1(AUC)×1.414, and φ−1 is the inverse of standard cumulative normal distribution.

In a similar study conducted by Javvaji et al., AUC for GCS and FOUR scores were compared [5]. They stratified the cases into "stroke" and "non-stroke" cases. AUC for GCS and FOUR were 0.75 and 0.85, respectively (correct to 2 decimal places), in non-stroke cases [5]. So, using the above formulae, 

V(AUC1)=V(AUC for GCS)=V(0.75)=0.1348; 

V(AUC2)=V(AUC for FOUR)=V(0.85)=0.09776; 

Under the null hypothesis (H₀):

\[ V_{H_0}(\widehat{AUC}_1 - \widehat{AUC}_2) = 2 \times V(\text{average of } AUC_1 \text{ and } AUC_2) \]

\[ V_{H_0}(\widehat{AUC}_1 - \widehat{AUC}_2) = 2 \times V(0.8) = 2 \times 0.11946 = 0.23892 \]

Under the alternative hypothesis (H₁):

\[ V_{H_1}(\widehat{AUC}_1 - \widehat{AUC}_2) = V(AUC_1) + V(AUC_2) - 2 \times r \times \sqrt{V(AUC_1)} \times \sqrt{V(AUC_2)} \]

Assuming $r = 0.5$:

\[ V_{H_1}(\widehat{AUC}_1 - \widehat{AUC}_2) = 0.11777 \]

Plugging in these values in the first equation, 

$n = \frac{(1.96 \times 0.48879 + 0.84 \times 0.34317)^2}{(0.75 - 0.85)^2}$
 

Thus, the sample size, n=1.5532/0.01=155.32=156 (approximately) for each of the "survived" and "mortality" groups. This value is consistent with the table of sample size provided by Hajian-Tilaki [30]. Hence, the total sample size=156+156=312.
